# Event and Comment

**Published:** 1906-12-15

**Authors:** 


THE
### DENTAL REGISTER.

Vol. LX. December 15, 1906. No. 12.

### EVENT AND COMMENT.

### Columbus Library.

At the recent meeting of the Ohio State Dental Society, through its officers, a report was made as to the condition of the Columbus Library. The officers of the Columbus Library Association have secured a fine start towards, what promises to be one of the best dental libraries in the country.

They have purchased from Dr. A. H. Fuller of St. Louis, his collection of Dental Journals and text books, which contains complete files of some of the oldest Dental Journals.

A strong appeal has been made to every practitioner in Ohio to contribute any books, which he may have that would be valuable in such a library. In response a large number of valuable contributions have already been secured, and more are promised.

The library will be housed in the beautiful new Columbus City Library, which has just been completed at a cost of over $250,000.00. The dental library will be securely housed and cared for under the rules of the City Library.

This is a most commendable project and should be helped on by everyone who can. The Ohio State Dental Society at the recent meeting contributed $300 to assist in the purchase of books, and the Columbus Dentists have contributed five hundred dollars.

**Ohio State Dental Society.**

The annual meeting of this Society was held at Columbus, Dec. 4th to 6th, and was one of the most interesting meetings ever held. There was an unusually large attendance and the program was a most attractive one. The papers were of great merit and discussed with great interest. If one were inclined to criticise, it would be to complain that there was too much work crowded into one meeting. There were five sessions devoted to the reading of papers and discussions, and two forenoons given over to clinics which were very attractive and interesting. Nearly thirty clinics were given each day. The displays by the dealers were very attractive, and seemed to meet the requirements of some who spent more time and money on them, than they do on more substantial matters.

The meeting was held in the Great Southern Hotel, which gave fairly good accommodations. The clinic and display rooms for the dealers were all that could be asked, but the hall used for meeting purposes was bad, the ventilation was poor and means of lighting very unsatisfactory.

We shall publish a sketch report of the proceedings in the near future. Dr. H. C. Brown, of Columbus was elected president; Dr. C. J. Keely, of Hamilton, Vice-President; Dr. W. A. Price of Cleveland, Treasurer, and Dr. F. R. Chapman, of Columbus, Secretary.

